# Sub-100 nm resolution microscopy based on proximity projection grating scheme

**DOI:** 10.1038/srep08589

**Published:** 2015-02-26

**Authors:** Feng Hu, Michael G. Somekh, Darren J. Albutt, Kevin Webb, Emilia Moradi, Chung W. See

**Affiliations:** 1 Department of Electrical and Electronic Engineering, University of Nottingham, Nottingham, NG7 2RD, UK; 2 Institute of Biophysics, Imaging and Optical Science (IBIOS), University of Nottingham, Nottingham, NG7 2RD, UK

## Abstract

Structured illumination microscopy (SIM) has been widely used in life science imaging applications. The maximum resolution improvement of SIM, compared to conventional bright field system is a factor of 2. Here we present an approach to structured illumination microscopy using the proximity projection grating scheme (PPGS), which has the ability to further enhance the SIM resolution without invoking any nonlinearity response from the sample. With the PPGS-based SIM, sub-100 nm resolution has been obtained experimentally, and results corresponding to 2.4 times resolution improvement are presented. Furthermore, it will be shown that an improvement of greater than 3 times can be achieved.

Optical microscopy is the mainstay of imaging in the biological sciences, on account of its relatively non-invasive nature and its ability to image live cells. The lateral resolution is, however, poor compared with techniques such as electronic microscopy, so there is a strong impetus to develop methods capable of imaging well beyond the diffraction limit of half an optical wavelength in order to visualise sub-cellular structures in tissue. In order to overcome the resolution limit of conventional optical microscopy, several super resolution microscopy techniques have been developed. These include structured illumination microscopy (SIM)^1^,^2^, stimulated emission depletion microscopy (STED)^3^,^4^ and stochastic optical reconstruction microscopy (STORM)^5^,^6^ or photoactivated localization microscopy (PALM)^7^. STED microscopy can achieve a lateral resolution of around 10 nm^8^,^9^,^10^. In order to obtain such a resolution, high intensity illumination is required, which may lead to photobleaching and increase the risk of sample damage. The lateral resolution of STORM or PALM can also reach the level of around 10 nm^11^,^12^. In order to reconstruct the high resolution image of a whole sample, a large number of images need to be captured, which limits its imaging speed.

The layout of a SIM is similar to that of a bright field optical microscope and therefore can be constructed as an add on. The additional optical components are used for illumination to project the optical grating onto the sample. By phase shifting the projected grating, and capturing the image at each phase shift, the sample can be reconstructed with an improved resolution. Compared with STORM or PALM, SIM requires fewer raw images. Thus, the imaging speed of SIM is potentially faster than STORM or PALM. Therefore, SIM has been widely used to obtain 3D images of live samples^13^,^14^. In addition, SIM does not require high illumination intensity, and thus reduces the possibility of photobleaching. The major disadvantage of SIM is that its resolution is still limited by diffraction and the maximum resolution improvement is a factor of 2 compared to conventional fluorescence optical microscopy. In order to improve the resolution of SIM further, nonlinear SIM (NSIM)^15^ or saturated patterned excitation microscopy (SPEM)^16^ have been proposed. However, these techniques employ nonlinear saturation effects in fluorophores, which can lead to severe photobleaching and has therefore restricted application in biological imaging^17^.

In this paper, we will use the proximity projection grating scheme (PPGS) to further enhance the resolution of a SIM. With this method, the structured illumination is generated using the PPGS. Similar to the conventional SIM, the sample illumination can be considered as consisting of a set of discrete harmonics whose k-vectors are dependent on the original grating and the medium between the grating and the sample. With the PPGS scheme, the k-vectors of these harmonics can have values much higher than those afforded with a conventional SIM. These harmonics represent an increase in the imaging bandwidth of the system, and therefore can lead to much improved resolution. This represents the crucial difference between our system and the conventional SIM. Apart from that, they are identical in all other aspects. It should be pointed out that the main purpose of the paper is to describe the characteristics and merits of the system, especially regarding lateral resolution that can be achieved. A number of samples will be used to illustrate this aspect, and comparison will be made with respect to conventional optical microscope as well as SIM. Further research is required in terms of applications, and will be reported in future publications.

Figure 1 shows the principle of the PPGS-based SIM and the grating unit. It consists of the illumination optics, PPGS unit and the imaging optics. The illumination optics provides a uniform plane wave incident at the grating unit. It also performs angular scanning of the light beam, which would effect the phase shifting of the illumination pattern, required for the reconstruction of the final image.

Sandwiched between the grating and the sample is a layer of high refractive index optical thin film (Fig. 1(b)). The optical grating diffracts the incident illumination into various orders, after propagating through the optical film, they interfere and form an intensity pattern at the sample/thin film boundary. The use of a high refractive index material as the optical film allows the period of the grating to decrease in proportion. This will result in a finer intensity pattern illuminating the sample. It is this aspect of the illumination configuration that allows the system to achieve resolution improvement of greater than 2.

The grating layer used in this paper is shown in Fig. 1(c). It consists of an equilateral triangle array, formed by etching nanoholes at the vertices of the triangles through a thin layer of chromium. The choice of a triangular pattern, as compared with say a square one, is that the illumination gives a more uniform coverage in the spatial frequency domain and still maintains a relatively simple structure.

Figures 2(a) and Fig. 2(b) are two intensity patterns, formed experimentally at the top surface of the thin film. Microscope immersion oil was used as the thin film and the thicknesses were 25 μm for Fig. 2(a) and 32 μm for Fig. 2(b). Also shown are patterns obtained from simulation, using a Fourier modal method (FMM)^18^,^19^. The similarities between the two sets of results are excellent. It is also worth noting the rapid change of the patterns over a small propagation distance.

The potential of the proximity projection grating was reported as a proof of concept in our previous publications^20^,^21^. Here we will use the PPGS to develop a genuinely high resolution microscopy technique by using thin film of high refractive index, namely Arsenic Trisulphide (As_2_S_3_). With the newly-developed PPG structure, sub-100 nm resolution has been achieved experimentally with an objective NA of 1.3. We have also applied the technique to cell imaging to demonstrate its potential in life science.

## Results

**Figure 2:**
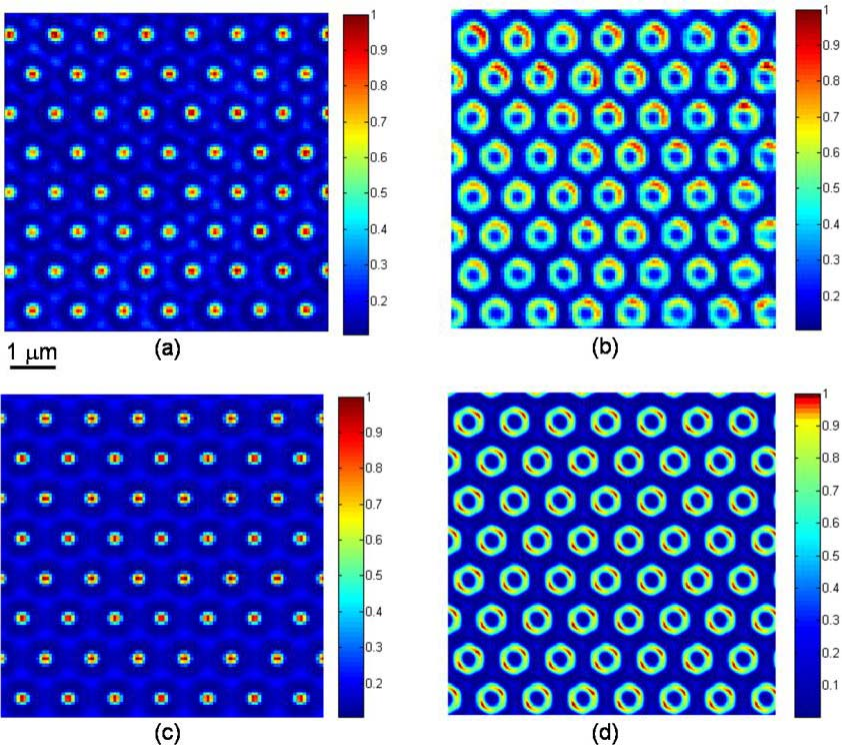
Diffraction intensity patterns of PPGS corresponding to different propagation distances (film thicknesses). (a) and (b): experimental results; (c) and (d): simulation results. Film thicknesses for (a) and (c): 25 μm; for (b) and (d): 32 μm.

Intensity patterns similar to those in Fig. 2 are used for sample illumination. These patterns play a vital role in the operation of the system. A properly selected pattern will allow high resolution imaging to be realized, and at the same time maintain the quality of the image obtained. This will impose certain requirements on the grating unit, in particular the shape of the grating and the refractive index of the optical thin film. Two grating units have been used to demonstrate these requirements. The specifications of the two are given in Supplementary Table S1 online.

**Figure 3:**
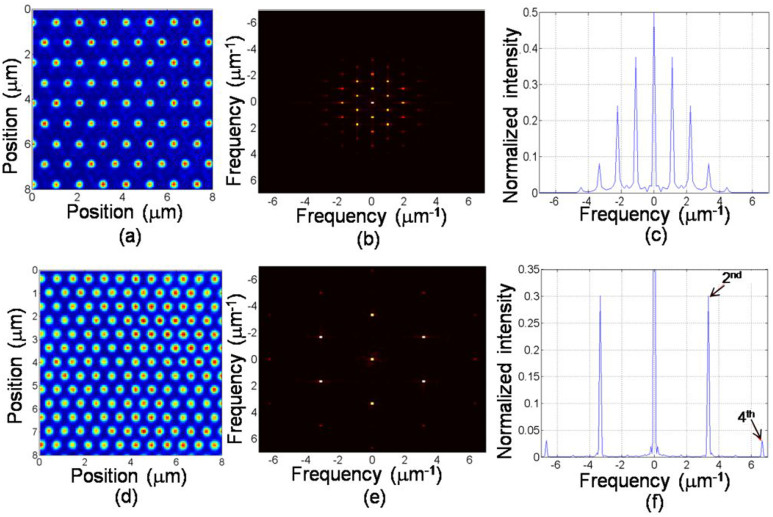
Illumination pattern used in the PPGS-based SIM and its spatial Fourier spectrum. (a) structured illumination from the PPGS with oil film; (b) 2D spatial Fourier spectrum of the structured illumination in (a); (c) 1D profiles along the vertical central position in (b); (d) structured illumination from the PPGS with As_2_S_3_ film; (e) 2D spatial Fourier spectrum of the structured illumination in (d); (f) 1D profiles along the vertical central position in (e).

Grating G1 is the one used to obtain the patterns shown in Fig. 2. Figure 3(a) shows an intensity pattern similar to that of Fig. 2(a). Figure 3(b) is the magnitude of the frequency spectrum of Fig. 3(a). Figure 3(c) is a line profile drawn vertically through the centre of Fig. 3(b). There are several points to note: 1) similar to SIM, the Fourier components in Fig. 3(b) are used to reconstruct the image of the sample. The lateral resolution of the final image depends on the high order components, in particular their magnitude compared to the dc term. In order to achieve good resolution, it is imperative that the intensity pattern should have as high a spatial frequency as possible. This can be achieved by using a grating of appropriate period. Ultimately, however, it is determined by the refractive index of the thin film used in the unit. A high refractive index material allows the k-vectors of the diffracted orders to have values beyond those that can be supported in air, and result in a fine intensity pattern. This is the key to realize super-high resolution performance^20^,^21^; 2) the second point concerns the number of Fourier components associated with the intensity pattern that are included in the reconstruction. The separation between any two adjacent frequency components should be smaller than the imaging bandwidth of the originally optical system. This will ensure the full spectrum is used for reconstruction. On the other hand, too many frequency components will slow down the system operation as the number of phase shifted images is proportional to the number of frequency components used; and 3) the Fourier components should be distributed uniformly in the frequency domain. As an example, a grating of triangular shape will result in a spectrum similar to the one in Fig. 3(b), which is preferred compared to the spectrum of a square grating, as the latter will have frequency components along the two orthogonal axes only, the information away from the axes will not be recovered.

Also shown in Fig. 3 are the intensity pattern and the frequency spectrum obtained using the second grating G2. With this grating, arsenic trisulphide (As_2_S_3_) is used as the thin film. As_2_S_3_ is a solid that has a refractive index of around 2.5 at λ ≈ 550 nm, and a transmission cut-off at around 500 nm^22^. It was deposited onto the grating using thermal evaporation, to a thickness of 15 μm. (The deposition of the As_2_S_3_ layer on the grating substrate was carried out at Technische Universität Kaiserslautern, Fachbereich Physik, Erwin Schrödinger Straße Geb. 56, 67663 Kaiserslautern, Germany.) As the period of G2 is 0.6 μm instead of 0.9 μm for G1, the frequency components of the grating G2 are higher (Fig. 3(c) and Fig. 3(f)). It is of interest to note that, because of the thickness of the As_2_S_3_ film used, both the first and third harmonics of the intensity pattern are suppressed. Changing the thickness of the thin film will change the relative magnitudes of the different harmonics. This point will be discussed further in a later section. From Fig. 3(c) and Fig. 3(f), the locations of the first order components for the two gratings are measured to be respectively 1.11 μm^−1^ and 1.67 μm^−1^. The ratio of the two is 1.5, which is the ratio of the two gratings periods.

In order to evaluate the resolution performance of the PPGS-based SIM, the system was used to image 40 nm diameter fluorescent beads (540/560 nm, INTROVIGEN). The configuration of the system was similar to that described in reference^21^ except high resolution imaging optics was used. The imaging lens was an Olympus 0.6–1.3 oil objective (UPLFLN × 100), and the grating G1 was used. As discussed in reference^21^, depending on the image reconstruction algorithm used, different imaging modes can be realised. These imaging modes include scanning optical microscope (SOM), scanning confocal microscope, SIM and PPGSIM. As they are derived from the same set of experimental result, they provide an ideal basis for comparing the resolution performance of various imaging modalities. In reference^21^, we have compared results obtained using PPGSIM to those corresponding confocal and SOM. In this paper we will investigate images reconstructed using different number of illumination harmonics, bearing in mind that image obtained using only the dc component in the reconstruction is identical to that obtained with a SOM. Furthermore, the best resolution improvement offered by the SIM, assuming an illumination NA equalling the imaging NA, is 2× that of a SOM. Comparisons will be made between our system and SOM and SIM on this basis.

**Figure 4:**
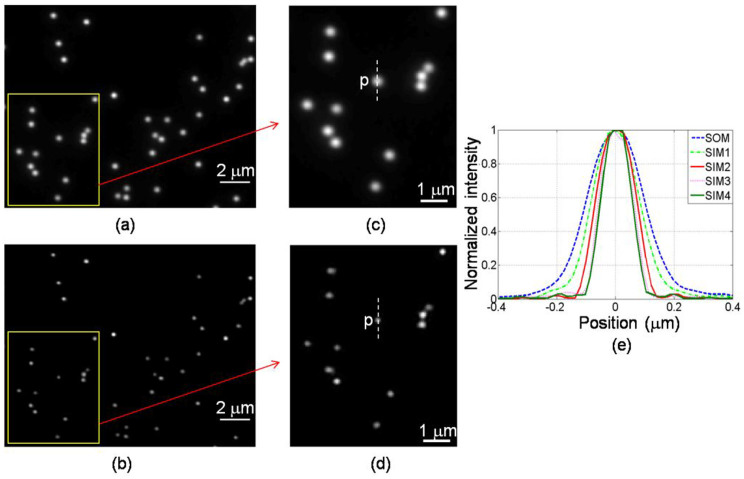
Images of fluorescent beads. (a) scanning optical microscopy (SOM, conventional); (b) PPGS-based SIM image with the 4th harmonic included in the reconstruction; (c) magnified images from the marked box in (a); (d) magnified images from the marked box in (b); (e) intensity profiles of the isolated bead ‘p' along the line indicated, with respect to different harmonics included in the reconstruction. SIM1-SIM4: the last digital number represents the harmonic included.

Figure 4(a) is the image of the fluorescent beads corresponding to the SOM and Fig. 4(c) is the enlarged version of the area shown inside the yellow box. Figures 4(b) and Fig. 4(d) are the equivalent images obtained using all four harmonics. The difference in the feature sizes of the two sets of results demonstrates the resolution improvement offered with the grating set up. Figure 4(e) shows the intensity profiles of the fluorescent bead marked ‘P' in Fig. 4(c). The profiles labelled SOM and SIM4 are from Fig. 4(c) and Fig. 4(d) respectively, and the other curves, labelled SIM1, SIM2 and SIM3 are reconstructed using 1, 2 and 3 harmonics. From Fig. 4(e), we could measure the FWHMs of the five responses. This exercise was repeated on 20 isolated beads and the resulting values were then averaged and given in Supplementary Table S2 online. Also included in the table are the effective imaging NAs required to achieve the FWHMs. The effective NAs are estimated using a scalar diffraction program, matching the targeted FWHM with the appropriate imaging NA, taking into account the diameter of the fluorescent beads^21^. The bottom row shows the resolution improvement of the different reconstruction methods, relative to the NA of the objective lens.

**Figure 5:**
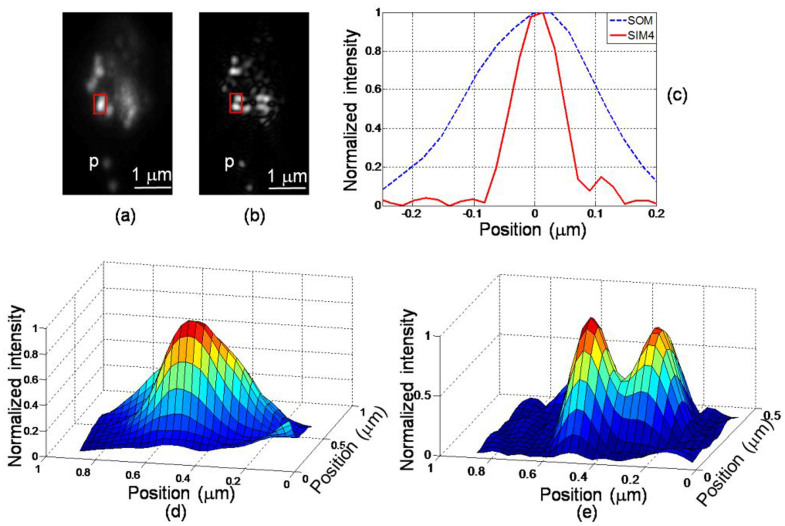
Images of fluorescent beads. (a) scanning optical microscopy (SOM, conventional); (b) PPGS-based SIM image with the 4^th^ harmonic included in the reconstruction; (c) intensity profiles of the isolated bead ‘p'; (d) intensity map from the marked box in (a); (e) intensity map from the marked box in (b).

The same experiment was repeated using grating G2. Figure 5(a) shows the SOM image of a small area of the sample, and Fig. 5(b) is the PPGS-based SIM image of the same area, in which the harmonics up to the fourth one were included in the reconstruction. Figures 5(d) and Fig. 5(e) are the intensity maps of the regions enclosed by the boxes in Fig. 5(a) and Fig. 5(b), showing the superior resolving power of the PPGS-based SIM system. Figure 5(c) shows the intensity line profiles of the particle marked P in Fig. 5(a) and Fig. 5(b). The FWHMs of the two profiles are 226 nm and 93 nm respectively, with the latter corresponding to an effective imaging NA of 3.03, or a resolution improvement of 2.4x.

**Figure 6:**
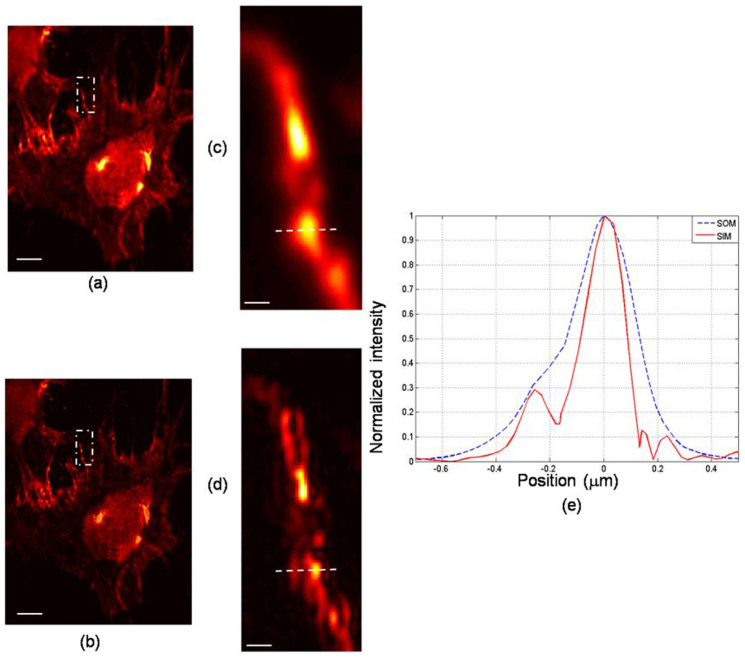
Fluorescent images of cells. (a) scanning optical microscopy (SOM, conventional); (b) PPGS-based SIM image; (c) magnified image in the marked box of (a); (d) magnified image in the marked box of (b); (e) intensity profiles along the lines indicated in (c) and (d). Scale bar: (a) and (b) 3 μm; (c) and (d) 0.45 μm.

**Figure 7:**
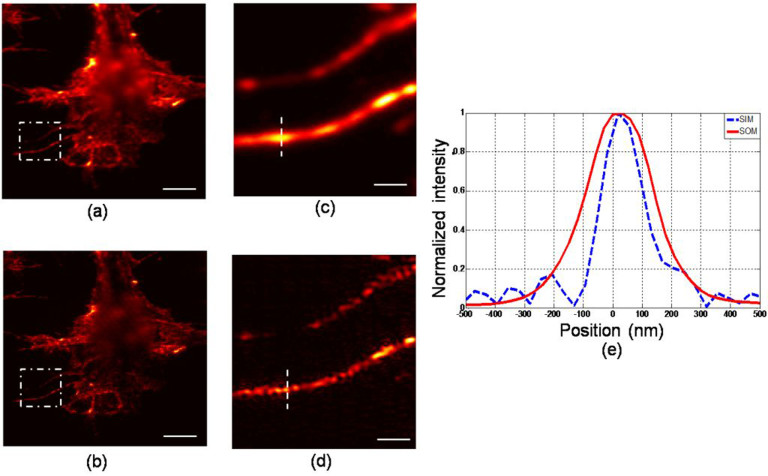
Fluorescent images of cells in another region. (a) scanning optical microscopy (SOM, conventional); (b) PPGS-based SIM image; (c) magnified image in the marked box of (a); (d) magnified image in the marked box of (b); (e) intensity profiles along the lines indicated in (c) and (d). Scale bar: (a) and (b) 3 μm; (c) and (d) 0.75 μm.

We will now present results obtained from biological samples using the PPGS-based SIM system. The sample used was a fibroblast cell from mouse embryonic (3T3 cells). The cells were grown on a coverglass and then fixed using fixation solution. The cells were stained with quantum dots in order to facilitate fluorescent imaging (see the Method section for details). The grating unit used for the experiment was G1. Figures 6(a) and Fig. 6(b) show the fluorescent images of the sample, reconstructed using the SOM and the PPGS-based SIM modes with 3 harmonics included. The regions enclosed by the boxes have been enlarged and shown in Fig. 6(c) and Fig. 6(d), again demonstrating the resolution improvement of the SIM technique. The intensity profiles along the dash lines in Fig. 6(c) and Fig. 6(d) were plotted in Fig. 6(e). In Fig. 6(e), a small feature can be seen appearing at the left of the mainlobe. The FWHMs for the spot features are 273 nm and 175 nm respectively. Compared with the conventional microscopy, the spot size has been greatly reduced in the PPGS-based SIM image. Figure 7 shows another set of images of the same sample. From Fig. 7(e), the FWHM of the red curve is estimated to be 267 nm, whereas the blue one is 165 nm. This demonstrates the resolution potential of the technique.

## Discussion

The lateral resolution of a SIM microscope depends on the extent of the spatial frequency components of the illumination pattern, which in turns depends on the period of the grating and the refractive index of the thin film. Similar to the conventional SIM, the PPGS based system described here is also limited by the spatial bandwidth of the illumination of the system. The advantage of the current method arises from the simple fact that the illumination medium is filled with a high index material, and is therefore akin to a solid immersion set up^23^. The configuration of the grating projection unit can afford further benefits. It is compact and can act as the sample substrate. By changing the wavelength or the profile of the incident light beam, or the thickness of the thin film, the illumination pattern can be altered drastically. This may facilitate different imaging modes as described elsewhere^21^.

A comparison can be made between the resolution performance of the experimental data and theoretical value. The former is obtained from results shown above. The theoretical resolution of the system depends on the refractive index *n* of the thin film material, the period of the grating, the highest frequency harmonics used in the reconstruction, and the numerical aperture *NA* of the original system. Based on these considerations, we can define an effective imaging numerical aperture, *NA_eff_*, for the PPGS SIM system. From the reference^20^, *NA_eff_* is given by
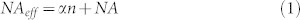
In the above equation, α is a scaling factor with value ranges from 0 to 1, and is dependent on the highest harmonic used in the reconstruction, which in turns depends on the angles of the diffracted orders from the grating. For example if the angle θ_i_ between two diffracted orders is 180°, then the α value will be 1. The best resolution the system can achieve, for any particular thin film material, is therefore *n* + *NA*. At the other extreme, if θ_i_ = 0, we will be back to the situation where the system will perform like an SOM.

The results shown in Fig. 5 are reconstructed using up to the 4^th^ harmonic. The corresponding α value is 0.7. With *n* = 2.5 and *NA* = 1.3, the effective system *NA* is therefore *NA_eff_* = 3.05. If we optimise the grating period so that the α value is nearly one, the effective *NA* would be *NA_eff_* = 3.8. Returning to the experiment with *NA_eff_* = 3.05, the diameter of the point spread function is 220 nm. The FWHM is 42% of the PSF's diameter, or 92 nm, which matches exactly the experimental value shown in Fig. 5.

The cell images shown above were obtained using oil film in the PPGS unit. In Fig. 5, we have presented results on fluorescent beads with As_2_S_3_ as the optical film, which showed appropriate improvement in resolution. Unfortunately it transpires that As_2_S_3_ was not compatible with the cells under examination, causing them to dehydrate and deform. Other high index materials, such as titanium dioxide (TiO_2_) or gallium phosphide (GaP) can be used as thin film and should not give rise to similar problem. The refractive indices of these is around 2.5 and 3.5 respectively, and their use in the present system will be investigated and presented in a future publication.

Another aspect of the system that requires further investigation concerns noise in the system. Of particular interest is the effect of noise on the reconstruction of the high spatial frequency features, as the magnitudes of the high harmonics tend to have lower values.

In conclusion, we have experimentally demonstrated a high resolution imaging system based on the proximity projection grating scheme. Sub-100 nm resolution has been achieved with a 2.4 times improvement in resolution compared with the conventional microscopy. The resolution can be further enhanced by optimising the grating period and by employing higher refractive index material as the thin film in the proximity grating unit. The proposed method will provide us with a path to overcome the resolution improvement limit of the conventional SIM without using any nonlinearity of fluorophores.

## Methods

### System setup and data acquisition

The schematic diagram of the whole system was given in Supplementary Fig. S1 online. The light source was a frequency doubled Nd:YAG laser (BWN-532-20E, B&W TEK* INC.). The lenses L1 and L2 (f1 = 110 mm, f2 = 30 mm) are combined to reduce the laser beam diameter and increase the illumination intensity. The laser and lens L1 were mounted on a 2D scanning stage (M112.1DG Physik Instrumente). Scanning the unit would cause the laser beam emerged from lens L2 to change in angle, which eventually would shift the intensity pattern at the top surface of the grating unit, thus satisfying the phase stepping process required for the image reconstruction. The photons emitted from the sample were collected using an objective lens (UPLFLN 100xOI2, Olympus). The final image, with a magnification of ×111, was captured using an EMCCD camera (iXon DU-885K, ANDOR TECHNOLOGY). The equivalent pixel size at the sample plane was 72 nm × 72 nm. An optical bandpass filter (Z488/532 nm, Chroma Technology Corp.) placed in front of the camera would transmit the desired wavelength. In order to reconstruct a high resolution image, the illumination intensity pattern was scanned in the x-y directions. The image at each scan point was then captured for subsequent processing. The entire operation of the system was controlled using a PC through a customized Labview program. The recorded images were processed in the Matlab environment according to the image reconstruction algorithm described below.

### Imaging reconstruction

The reconstruction procedure was the same as that used in a conventional SIM^1^,^14^, with the reconstruction performed in the 2D spatial frequency domain. Additionally, considering the changes of both amplitudes and phases of the structured illumination, the reconstruction matrix in equation (2) needs to be employed.
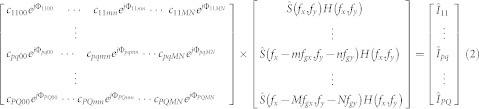
where Φ*_pqmn_* and *c_pqmn_* represent the phase and magnitude for the harmonic (*m*,*n*) at the scanning position (*p*,*q*). The phase includes the initial phase of the structured illumination as well as one induced by phase shift. *M* and *N* are the harmonics along x and y directions (*m = −M*: *M*,*n* = −*N*:*N*). 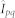 is the Fourier transform of the recorded fluorescence image at the scanning position (*p*,*q*). *H*(*f_x_*, *f_y_*) is the optical transfer function of system and 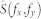 is the Fourier spectrum of sample. After all data were obtained, the information of the first and last matrix terms are known. Thus, the unknown components in the second matrix can be solved by the pseudo-inverse technique. After all contributions are obtained, the remaining process is the same as the conventional SIM.

### Fluorescent beads sample preparation

The fluorescent beads used in the quantitative analysis of the system resolution have 40 nm diameters (540/560 nm, INTROVIGEN). For the experiments based on the PPGS with oil film, a drop of diluted solution was deposited on a coverglass. Then, the coverglass needed to be put in a warm place for 30 minutes until the diluted bead solution became absolutely dry. Finally, a thin layer of immersion oil was sandwiched between the sample substrate and grating G1. For the experiments based on the PPGS with the As_2_S_3_ film, a layer of fluorescent beads were deposited directly onto the top surface of the As_2_S_3_ film for imaging.

### Cell sample preparation

3T3 cells (used between passages 35–38) were obtained from American Type Culture Collection (ATCC) and were grown to confluence in 75 cm2 flasks in Dulbecco's Modified Eagle Medium (DMEM) (Sigma-Aldrich, UK), supplemented with penicillin (100 units/ml), streptomycin (0.1 mg ml^−1^), amphotericin B (0.25 μg ml^−1^), Fetal Calf Serum (FCS) (10%) and L-glutamine (2 mM). Upon confluence, cells were detached from the flasks and seeded on coverslips at a seeding density of 10^4^ and were maintained at 5% CO2, 37°C overnight.

Cells were then fixed in 4% w/v paraformaldehyde (diluted in PBS) for 15 min at room temperature and permeabilised by incubating with Triton X-100 (0.1% v/v in PBS), followed by PBS washes. Actin filaments were then labelled with quantum dots (Qdot®655 streptavidin conjugate, Life Technologies), using the provided protocol. Briefly, permeabilised cells were incubated first with an actin specific phalloidin-biotin conjugate (Sigma-Aldrich, UK) in PBS for 60 min to provide binding sites for the quantum dots, followed by washing in PBS. The cells were then incubated with the quantum dot-streptavidin conjugate for 60 min to label the actin filaments, followed by washing in PBS.

## Author Contributions

C.W.S. and M.G.S. conceived the idea. F.H. implemented the experiments and processed the data. D.A., K.W. and E.M. specified, prepared the biological samples and interpreted the results. C.W.S., F.H. and M.G.S. were responsible for the manuscript. All authors reviewed the manuscript.

## Supplementary Material

Supplementary Information Sub-100 nm resolution microscopy based on proximity projection grating scheme

## Figures and Tables

**Figure 1:**
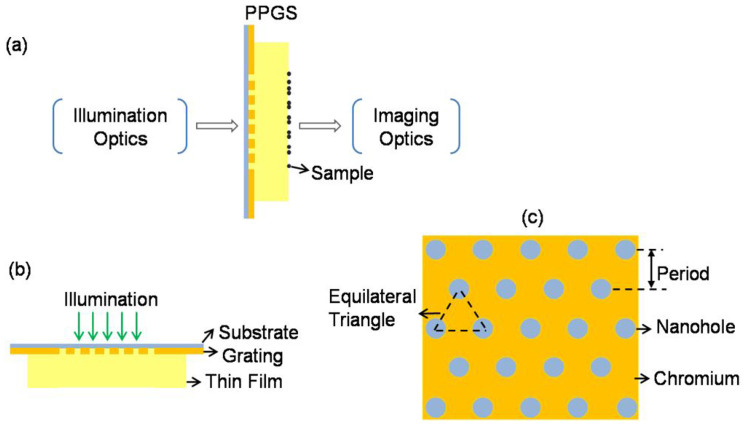
Principle of PPGS-based SIM. (a) schematic diagram of the proposed method; (b) proximity projection grating scheme (PPGS); (c) hexagonal nanohole array grating.
