# Supplementary material for: Sub-100 nm resolution microscopy based on proximity projection grating scheme
Source: Sci Rep. 2015 Feb 26;5:8589. doi: 10.1038/srep08589 (PMC4341199; doi:10.1038/srep08589)
Supplement: Supplementary Information — Sub-100 nm resolution microscopy based on proximity projection grating scheme [file srep08589-s1.pdf]

## Supplementary Information

# Sub-100 nm resolution microscopy based on proximity projection grating scheme

Feng Hu[1], Michael G. Somekh[2], Darren J. Albutt[2], Kevin Webb[2], Emilia Moradi[2], and Chung W. See,[1,*]

[1]Department of Electrical and Electronic Engineering, University of Nottingham, Nottingham, NG7 2RD, UK

[2]Institute of Imaging and Optical Science (IBIOS), University of Nottingham, Nottingham, NG7 2RD, UK

*chung.see@nottingham.ac.uk

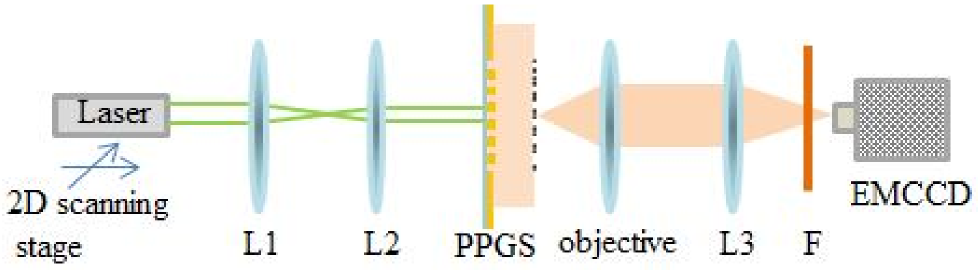


Supplementary Figure S1. System setup of PPGS-based SIM. L1, L2: lenses with focal length 110 mm and 30 mm, respectively. Objective: NA 1.3, oil immersion. L3: tube lens with focal length 200 mm. F: emission filter. EMCCD: Electron Multiplying Charge Coupled Device.

Supplementary Table S1 Parameters of grating units used in the experiment

| | Grating period | Hole diameter | Thin film material | Thin film refractive index | Thin film thickness |
|---|---|---|---|---|---|
| Grating G1 | 0.9 μm | 300 nm | Immersion oil | 1.51 | 25 μm |
| Grating G2 | 0.6 μm | 200 nm | As2S3 | 2.5 | 15 μm |

Supplementary Table S2 FWHM measured using five different reconstruction methods

| | SOM | SIM1 | SIM2 | SIM3 | SIM4 |
|---|---|---|---|---|---|
| FWHM in nm | 222±2 | 185±4 | 156±4 | 133±4 | 120±4 |
| Effective NA | 1.27 | 1.52 | 1.81 | 2.12 | 2.35 |
| Resolution improvement | 1x | 1.20x | 1.43x | 1.67x | 1.85x |
